# The Effect of Edge Mode on Mass Sensing for Strained Graphene Resonators

**DOI:** 10.3390/mi12020189

**Published:** 2021-02-12

**Authors:** Xing Xiao, Shang-Chun Fan, Cheng Li

**Affiliations:** 1 School of Instrumentation and Optoelectronic Engineering, Beihang University, Beijing 100191, China; 2 Key Laboratory of Quantum Sensing Technology (Beihang University), Ministry of Industry and Information Technology, Beijing 100191, China; 3 Shenzhen Institute of Beihang University, Shenzhen 518063, China

**Keywords:** graphene resonator, edge mode, mass sensing, quality factor, molecular dynamics simulation

## Abstract

Edge mode could disturb the ultra-subtle mass detection for graphene resonators. Herein, classical molecular dynamics simulations are performed to investigate the effect of edge mode on mass sensing for a doubly clamped strained graphene resonator. Compared with the fundamental mode, the localized vibration of edge mode shows a lower frequency with a constant frequency gap of 32.6 GHz, despite the mutable inner stress ranging from 10 to 50 GPa. Furthermore, the resonant frequency of edge mode is found to be insensitive to centrally located adsorbed mass, while the frequency of the fundamental mode decreases linearly with increasing adsorbates. Thus, a mass determination method using the difference of these two modes is proposed to reduce interferences for robust mass measurement. Moreover, molecular dynamics simulations demonstrate that a stronger prestress or a higher width–length ratio of about 0.8 could increase the low-quality factor induced by edge mode, thus improving the performance in mass sensing for graphene resonators.

## 1. Introduction

Since Geim and co-workers fabricated monolayer graphene by mechanical exfoliation in 2004 [1], graphene-based device manufacturing infrastructure has seen considerable advancements [2,3,4,5] due to the excellent mechanical properties [6,7,8], preeminent thermal properties [9,10], and tunable electrical performances [11,12,13,14]. A variety of nanotechnologies have been developed, including thermal interface materials [15,16,17]; electromagnetic composites [18,19]; gas separation membranes [20]; and sensor-based applications [21], such as accelerometers [22], biomedical detection [23], and gas measuring [24]. Among them, the graphene-based mass sensor has attracted increasing attention with the benefit of its unprecedented atom-thick two-dimensional structure, which provides enough area for the incoming mass flux. Traditional graphene mass sensors mainly operate by means of the conductance changes they cause to the adsorbed mass [24,25,26,27], which is susceptible to temperature variation and is only suitable for certain gases. However, the manufacture of the graphene resonator in 2007 [2] provided an alternative approach for mass sensing with the aid of frequency change induced by the adsorbent mass, thereby representing a promising method to achieve atomic-scale resolution.

To date, a number of authors have studied various methods to realize atomic-scale mass sensing by using graphene resonators. For example, Duan et al. [28] adopted a hybrid structure of graphene sheets supported by carbon nanotubes as a resonator, and then performed molecular dynamics (MD) simulations to demonstrate ultrahigh mass resolution up to 1 yg (10^−24^ g) with a mass responsivity of 0.34 GHz/yg. Then, Jiang et al. [29] investigated the feasibility of the graphene nanomechanical resonant mass sensor and showed that the mass sensitivity would triple if the actuation energy was about 2.5 times the initial kinetic energy of the nanoresonator. In addition, some efforts, including decreasing geometric sizes [30], utilizing vacancies [31], and adjusting capacitive force [32], have been made to improve the performance of graphene-based resonant mass sensors. Though ultrahigh mass resolution of up to 1 yg has been reported, the impact of edge modes, which always occur at the free edges of graphene sheets and even break the coherence of fundamental oscillation [3,33], has rarely been discussed.

The molecular dynamics method, which directly computes the state of every atom and takes the scale effect into consideration, seems to be the most suitable method to investigate the edge mode of graphene sheets compared with finite element methods [34], nonlocal elasticity theory [35,36], and molecular structural mechanics methods [37]. As a computer simulation method for analyzing the movements of atoms and molecules and providing a view of the dynamic “evolution” of the studied system, molecular dynamics has been used to study the mechanical properties [38,39] and thermal properties [40,41,42] of novel nanomaterials including graphene, and the results meet well with those of the experiment.

In this paper, molecular dynamics simulation is performed to investigate the distinct responses between edge modes and fundamental modes to adsorbed mass on graphene sheets, and a novel mass determination method based on the frequencies of these two modes is proposed, which is expected to diminish the interferences caused by mutable stress in graphene. In previous studies, the mass was determined by the fundamental frequency shift, which is hypersensitive to the stress in graphene in addition to the absorbed mass. However, it is difficult to control the stress in graphene accurately and steadily. For example, temperature fluctuation can induce distinct thermal expansions between graphene and silicon oxide substrate, thereby changing the graphene inner stress significantly [2]. Moreover, the shift in gate voltage, if actuated using electrical methods, can disturb the stress [4]. MD simulations in our study indicate that the frequency difference between fundamental eigenmodes and edge eigenmodes shows a faint dependency upon stress variance. Hence, adopting edge mode in mass determination is expected to minimize the disturbance of stress fluctuations and identify the absorbed mass more accurately. In this case, in combination with the negative effects [33,43] induced by edge modes on the quality factor of the graphene resonator, proper improvement methods, including exerting stronger prepress and maintaining a lager width–length ratio, are proposed to ensure the mass sensing performance of graphene resonators with edge modes.

## 2. Simulation Structures and Methods

Molecular dynamics simulations have been used to study the impact of edge modes on mass sensing for strained graphene resonators [44,45]. The studied doubly clamped graphene sheets with adsorbates are commonly treated as divided atoms and molecules interacted by interatomic potentials or molecular mechanics force fields [46,47,48]. For the systems obeying the ergodic hypothesis, the macroscopic thermodynamic properties can be confirmed by directly tracing the trajectories of atoms and molecules, usually by means of numerically solving the corresponding Newton’s or Hamilton’s equations.

**Figure 1:**
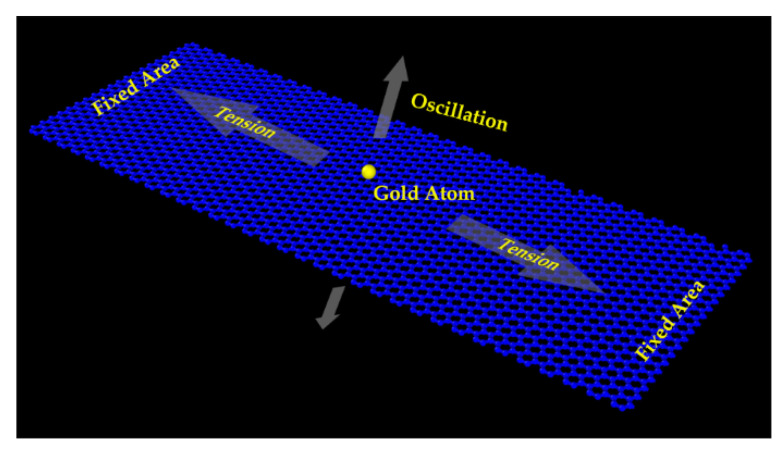
Atomic schematic of doubly clamped monolayer graphene resonator for classic molecular dynamics (MD) simulation.

In the present MD simulation, the modeling schematic diagram of a graphene-based resonant mass sensor is shown in Figure 1. The sensing element as a nanoscale resonator is a doubly clamped monolayer graphene sheet. Under the actuation imposed by external force (either electrical or optical method), the vibration frequency of graphene sheet depends largely on the absorbed mass and graphene inner tension. Note that if the tension is kept steady or the tension-reduced frequency shift is compensated appropriately, absorbed mass can thus be deduced from the graphene sheet’s mechanical frequency.

In Figure 1, graphene sheets with a length of 100 Å and widths ranging from 50 Å to 100 Å, composed of 1862–3795 carbon atoms, were considered, and on the surface, one or more gold atoms were added as absorbed mass. The interactions among these atoms were described using three widely used potential functions. Covalent bonds between carbon atoms were expressed as the adaptive intermolecular reactive empirical bond order (AIREBO) [47,48,49], while the potential and embedded-atom method (EAM) [50] was used to describe the interactions among multiple gold atoms. Then van der Waals adhesion energy between graphene and absorbed gold atoms was estimated using the Lennard–Jones 12-6 equation [51]. All of the MD simulations were performed with the help of the Large-scale Atomic/Molecular Massively Parallel Simulator (LAMMPS) package from Sandia National Laboratories (Albuquerque, NM, USA) [45]. In this case, the developed MD simulation model as indicated in Figure 1 underwent a 100 ps equilibration process with a time step of 1 fs under the NPT ensemble, where the number of atoms, the pressure (0 Pa), and the temperature (10 K) were kept constant in order to obtain the relaxed structure with minimum energy. Then, a moderate and continuous deformation was imposed to exert axial stress ranging from 10 to 50 GPa. The strained graphene sheet was doubly fixed and underwent a second equilibrium process. Afterwards, a sinusoidal initial velocity profile was applied to the graphene sheet to excite flexural vibration, and the initial velocity was constrained under 1 Å/ps to avoid violent nonlinear vibration according to our previous work [52]. In this way, the doubly clamped graphene sheet started vibrating under the NVE ensemble, where the atom numbers, the volume, and the energy were constant. During the simulation period, essential physical quantities, including kinetic energy, potential energy, stress, and oscillation amplitudes, were recorded for further frequency response analysis.

## 3. Results and Discussions

### 3.1. Two Distinct Vibration Eigenmodes

Referring to the previous literature [4,6,53], doubly clamped monolayer graphene sheets can be regarded as tensioned membranes without bending stiffness. Thus, according to the continuum elastics model, the corresponding fundamental resonant frequency is expressed as

(1) $$f_{fundamental}=\frac{1}{2L}\sqrt{\frac{\sigma }{\rho }}$$

where *L* is the length of the graphene sheet and *σ* is the axial tension in graphene. The equivalent density *ρ*, in terms of the contributions from both graphene and adsorbates, is calculated considering the thickness of the monolayer graphene sheet to be 3.35 Å and the ideal density to be 2200 kg/m^3^ for pure grapheme [2,5].

**Figure 2:**
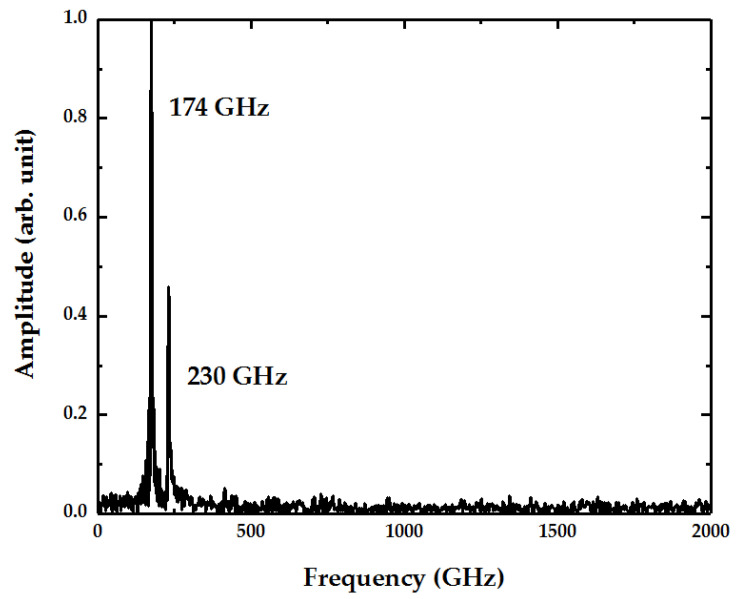
Normalized fundamental spectrum of kinetic energy for a doubly clamped graphene sheet with a length of 100 Å and a width of 50 Å.

As for the doubly clamped vibrating graphene sheet with a length of 100 Å and a width of 50 Å, as shown in Figure 1, an axial stress of 10 GPa was exerted on it with a gold atom placed in the middle as absorbed mass. Figure 2 illustrates the frequency spectrum of the kinetic energy obtained by MD simulation. Apparently, there are two peaks at 174 and 230 GHz, representing two distinct vibration eigenmodes, respectively. Theoretically, the fundamental mechanical frequency of the graphene sheet is deduced to be 117 GHz from Equation (1) by neglecting the effect of this gold atom. The corresponding frequency of the kinetic energy is 234 GHz, moving closer to the second peek in Figure 2, which is twice that of the mechanical resonant frequency. Consequently, it can be estimated that the second peek represents the fundamental frequency, while the first peek results from another vibration mode.

**Figure 3:**
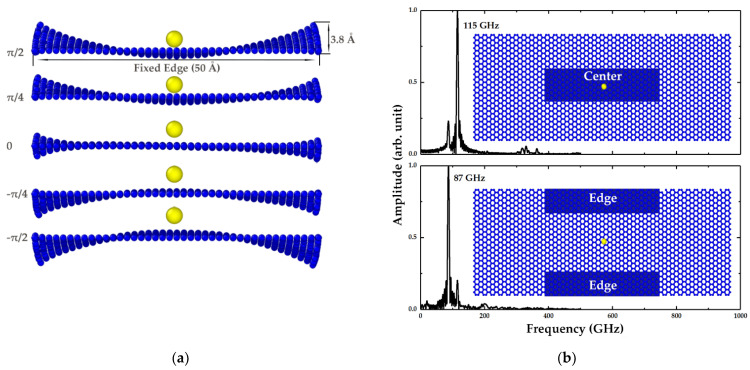
(**a**) Shape of edge eigenmodes. Lengths of fixed and free edges are 50 and 100 Å, respectively. Maximum out-of-plane displacement of the edge mode is 3.8 Å. (**b**) Normalized spectrums of average out-of-plane displacements of center group and edge group of carbon atoms, respectively.

To identify which vibration eigenmode the first peek represents, the mechanical vibrations of the graphene sheet were observed by visualization software OVITO [54], and not unexpectedly, two distinct eigenmodes were found. One eigenmode was the typical fundamental eigenmode illustrated above, while the other was localized at the free edges of the graphene sheet called “edge mode”, as depicted in Figure 3, where the local bulking induced by the edge modes has a lager out-of-plane displacement with an amplitude of 3.8 Å, just like the flapping wings. In contrast, the vibration amplitude in the fundamental mode was only about 1.5 Å. More importantly, the flapping movement had a different frequency to the fundamental vibration, thereby resulting in the two peaks in the kinetic energy spectrum, as shown in Figure 2. To verify this, the oscillation amplitudes of the center group and edge group of carbon atoms were recorded, respectively. Since the edge modes are located at the free edges of graphene sheet, the central part vibrated following the fundamental mode with a frequency of 115 GHz as presented in Figure 3b, where a small peak on the left represents the subtle effects of edge modes. At the free edges, since the edge mode plays a dominant role, the spectrum showed a peak at 87 GHz, which represents the edge mode frequency and corresponds to the first frequency peak in Figure 2. Moreover, it is worth mentioning that the vibration of the edge eigenmode was stronger than that of the fundamental eigenmode in this case; however, the proportions of the edge eigenmodes decreased quickly when exerting stronger stress or widening the graphene sheets.

### 3.2. Effect of Edge Mode on Mass Sensing

#### 3.2.1. Distinct Response to Centrally Distributed Adsorbates

Resonant mass sensors are dependent on the fundamental frequency shifts in response to adsorbates. When the external mass is absorbed on the graphene sheet, the equivalent density *ρ* will increases, thus reducing the fundamental frequency. The frequency shift can be written by

(2) $$\Delta f_{fundamental}=-\frac{1}{4L}K\sqrt{\frac{\sigma }{\rho_{0}}}\frac{\Delta \rho }{\rho_{0}}$$

where *ρ*_0_ is the density of pure graphene and ∆*ρ* is the increment of equivalent density, assuming the absorbed mass is evenly distributed. The factor *K* is used to adjust the frequency shifts caused by distinct distributions of adsorbates and equals 1 when adsorbates are evenly distributed. Since the volume of graphene sheet can be regarded to be unchanged, Equation (2) is then rewritten as

(3) $$f_{fundamental}=\left(\frac{1}{2}-\frac{K}{4}\frac{\Delta m}{m_{0}}\right)\frac{1}{L}\sqrt{\frac{\sigma }{\rho_{0}}}$$

where *m*_0_ is the mass of pure graphene sheets and ∆*m* represents the mass of adsorbates. In contrast, due to the unpredicted results when using standard elastic beam theory, the frequency of edge modes shows a considerably different response to central adsorbates.

**Figure 4:**
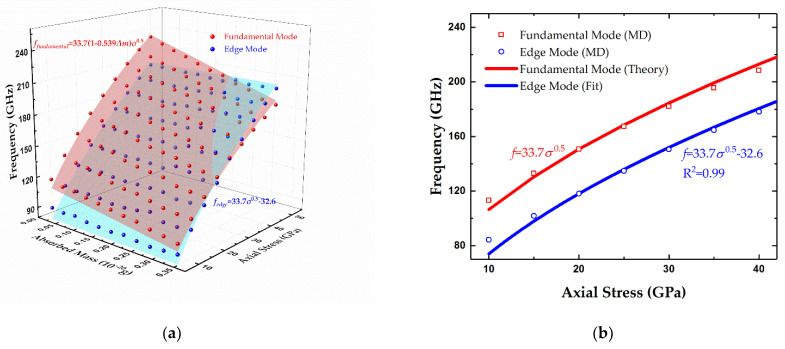

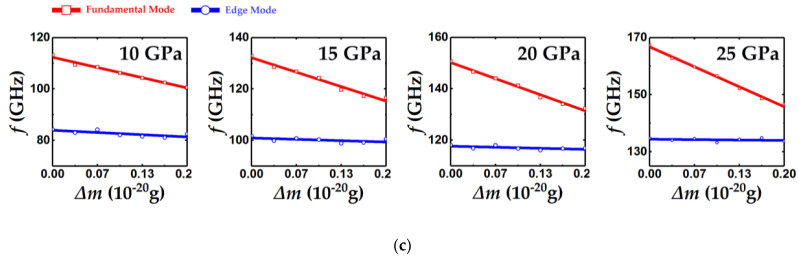
Resonant frequencies of fundamental and edge modes versus (**a**) centrally distributed absorbed mass and axial stress, (**b**) axial stress with no adsorbates, and (**c**) absorbed mass the axial stress in graphene sheet ranging from 10 to 25 GPa. Length and width of the doubly clamped graphene sheet are 100 and 50 Å, respectively.

To compare the distinct response to the centrally distributed absorbates between edge modes and fundamental modes, 1–10 gold atoms as adsorbates were placed in the middle of the graphene sheet with a length of 100 Å and a width of 50 Å, along with the applied axial stress ranging from 10 to 50 GPa. Figure 4a presents an overview of the resonant frequencies of two modes versus the mass of adsorbates and axial stress. This chart can be explained from several perspectives. Firstly, the resonant frequencies of the two modes show the significant dependence on axial stress, and they are both proportional to the square root of the stress. For clarity, the frequencies without adsorbates as a function of axial stress are displayed in Figure 4b. For the fundamental eigenmode, the theoretical result can be expressed as 33.7σ^0.5^ according to Equation (1), and it matches with the MD results considerably well, with an R-square of more than 0.99 and a maximum relative error of about 6%. Moreover, this relative error decreases to about 2% if a stronger tension (>15 GPa) is exerted, which can be explained by the fact that the stretched graphene sheet is closer to the ideal tensioned membrane structure. On the other hand, the two curves in Figure 4b reveal that the difference between fundamental frequency and edge frequency seems to remain steady despite the variational stress. Thus, the resonant frequency of edge modes can be described as

(4) $$f_{edge}=\frac{1}{2L}\sqrt{\frac{\sigma }{\rho_{0}}}-C_{bias}$$

where *C_bias_* is a constant. Equation (4) is then used to fit the MD results, showing an excellent R-square value up to 0.99, as displayed in Figure 4b, and the frequency gap between the fundamental modes and edge modes is about 32.6 GHz. From a different perspective, unlike the surface of fundamental modes warping along the axis of absorbate mass in the chart in Figure 4a, the edge modes’ fitting surface remained flat, thus revealing that edge modes are quite unsensitive to the centrally distributed adsorbates. To exhibit the relations between frequencies and adsorbed mass clearly, the sections of 10, 15, 20, and 25 GPa from Figure 4a were chosen and are individually presented in Figure 4c. It is apparent that the fundamental frequencies show a clear linear relation with the mass of attached gold atoms. Moreover, the further fitting results show the slopes of −60.9, −85.9, −96.0, and −107 with R-squares of 0.98, 0.98, 0.99, and 0.99, respectively. In contrast, the frequencies of edge modes show low variances of less than 4% in response to the absorbates. The corresponding R-squares are 0.43, 0.30, 0.36, and 0.17, respectively, which indicates weak linear relationships. As a result, the frequency of the edge mode still conforms to Equation (4) with the centrally distributed adsorbates.

#### 3.2.2. Determination of Centrally Distributed Mass in the Two Modes

The working principal of typical graphene-based resonant mass sensors complies with Equation (3), where the linear relationship between the fundamental frequency and adsorbate mass is critical for mass determination, assuming the stress σ in graphene is constant. However, the stress in graphene sheet is susceptible to numbers of factors, such as the temperature fluctuations [2], the variance of the gate voltage by electrical actuation [4], and the adsorbates themselves [3]. If the stress σ in Equation (3) is not a constant, the absorbed mass cannot be determined accurately simply by the fundamental frequency. Fortunately, the edge eigenmode may offer a solution.

The MD simulation results shown in Figure 4a indicate that when adsorbed mass is distributed at the center of graphene sheets, frequencies of fundamental eigenmodes and edge eigenmodes conform with Equation (3) (K equals 4 derived from MD results) and Equation (4). In this case, since the two independent equations regarding absorbate mass and stress are included, the absorbate mass and stress can be solved together, thus eliminating the interference induced by mutable stress. Then, by combining the frequencies of fundamental modes and edge modes expressed as Equations (3) and (4), the mass of central adsorbates can be defined by

(5) $$\Delta m=\frac{m_{0}}{2}\left(1-\frac{f_{fundamental}}{f_{edge}+C_{bias}}\right)$$

where ∆*m* is absorbed mass; *m*_0_ is the mass of the graphene sheet (excluding fixed areas); *C_bias_* is a constant as illustrated before, which is irrelevant to the stress in graphene. Note that the above derivation is under the premise that the mass is centrally distributed; nevertheless these adsorbates cannot be located at the exact middle of graphene sheets. Thus, the acceptable range of the adsorbates where the mass determination method proposed above is applicable is worth discussing.

**Figure 5:**
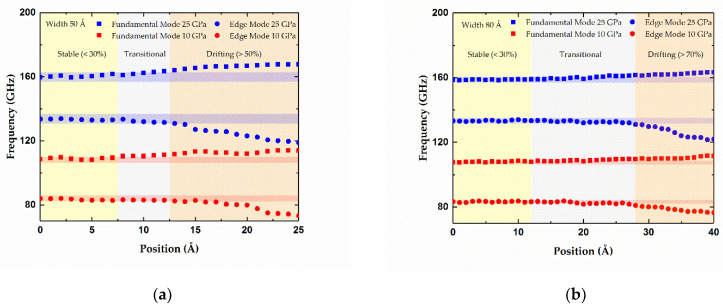
Resonant frequencies of the fundamental modes and edge modes versus the adsorbate positions along the width of graphene sheets. (**a**) Width of the graphene sheet is 50 Å. (**b**) Width of the graphene sheet is 80 Å.

To determine the appropriate range for centrally distributed adsorbates, two gold atoms as absorbed mass were moved from the center of graphene sheets to the edges along the width direction, and then the corresponding frequencies of the two modes were recorded and are depicted in Figure 5. Two samples of graphene sheet with sizes of 100 × 50 Å and 100 × 80 Å were considered, and different levels of stress, including 10 and 25 GPa, are discussed, respectively. Herein, the fundamental frequency shift induced by the single gold atom, which can be calculated by Equation (3), was chosen as a threshold. When the gold atoms move from the interior to the edge, if the resonant frequency drift is beyond this threshold, they can be regarded as out of central area, and the proposed mass determination method in Equation (5) would be unapplicable. Under this criteria, the adsorbates with a transversal distance within 30 percent of the width can be regarded as being in the middle, as shown in Figure 5, since the frequency drifts are less than the threshold. However, the adsorbates with a transversal distance of more than 50% of the graphene sheet with a 50 Å width and 70% of the graphene sheet with a 80 Å width, as displayed in Figure 5a and Figure 5b, respectively, lead to much higher frequency shifts than the threshold and then cannot be treated as being in the middle. Note that the wider graphene sheets tend to have narrower drifting parts where the resonant frequencies vary drastically. Thus, enlarging the width properly is an effective way to increase the valid center area for the added adsorbates.

#### 3.2.3. Constraints for Evenly Distributed Adsorbates

**Figure 6:**
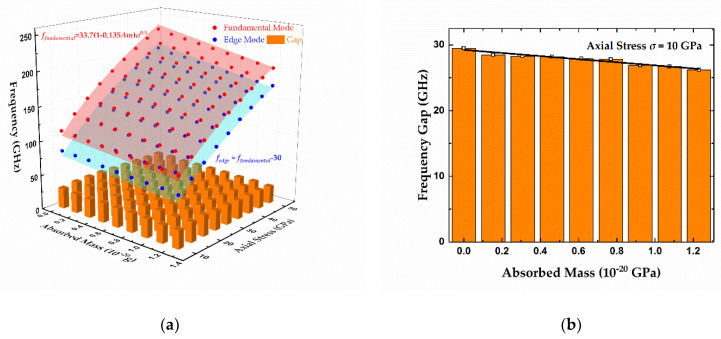
(**a**) Resonant frequencies of fundamental modes and edge modes versus evenly distributed absorbed mass and axial stress. Length and width of the considered doubly clamped graphene sheet are 100 and 50 Å, respectively. (**b**) Resonant frequency gap between fundamental modes and edge modes versus the absorbed mass with axial stress of 10 GPa.

The discussions and conclusions above make sense only in the case of centrally distributed adsorbates. When adsorbed mass is evenly distributed, the frequencies of the fundamental mode and edge mode decrease synchronously, as can be seen in Figure 6a, which exhibits the MD results (dots) and theoretical results (surfaces) of frequency shifts for both modes with evenly located adsorbates. The differences between the two eigenmodes remain almost unchanged with variational absorbed mass and stress, and the range of the frequency gap is within 4 GHz. Figure 6b provides the MD results under 10 GPa axial stress, which indicates that the frequency gap declines slightly (<4 GHz) with increased absorbates, and the linearity is not that strong, with a R-square of 0.91. As a result, the frequency gap between these two modes can be regarded as constant, and the relation can be described as *f_edge_* ≈ *f_fundamental_* − 30. In this case, we cannot obtain two independent equations regarding absorbate mass and stress and solve them together like in the case of central distribution. That is, the frequency of edge modes cannot provide more independent and valid information for mass determination, so the mass determination method proposed above is not applicable for evenly distributed adsorbates.

### 3.3. Effects of Edge Mode on the Q Factor

The quality factor (Q) is always an important issue for resonant sensors, and a higher Q factor means less energy dissipation per oscillation period and higher resolution for mass sensing [55,56]. The Q factor for graphene-based resonators has been measured to be 100–1800 at temperatures from 300 to 50 K since it was first reported [2], and recently, it was increased to 9000 when cooled down to 10 K [5]. Besides the intrinsic energy dissipation of graphene, extrinsic loss mechanisms, including air damping (if not operating in a vacuum) and clamping losses, are keys limiting factors [55]. In addition, edge mode is also a crucial mechanism causing a low Q factor [3,33], especially for doubly clamped graphene sheets.

**Figure 7:**
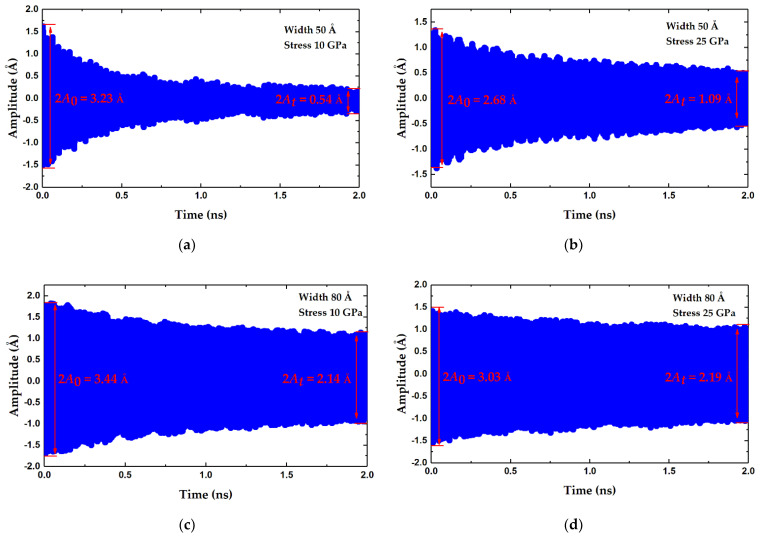

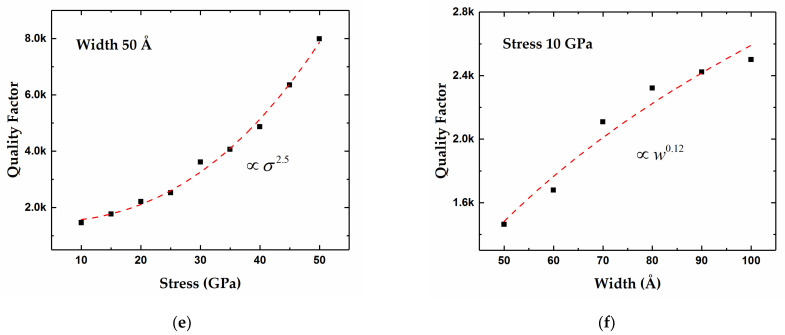
(**a**–**d**) Diminishing vibration amplitude with time for four graphene sheets with different widths and stress. (**a**) 50 Å, 10 GPa. (**b**) 50 Å, 25 GPa. (**c**) 80 Å, 10 GPa. (**d**) 80 Å, 25 GPa. (**e**) Q factor versus axial stress. (**f**) Q factor versus width.

To elucidate the edge effects on the Q factor for improving Q value, graphene sheets with different widths and inner stress were investigated by recording the diminishing amplitudes of fundamental vibrations that imply energy dissipations. Figure 7a–d compare the different amplitude damping of four samples. Since the square of amplitude is in direct proportion to the oscillation energy, the Q factor can be confirmed by

(6) $$Q=2\pi ft\frac{A_{0}^{2}}{A_{0}^{2}-A_{t}^{2}}$$

where *f* is the fundamental frequency; *t* is duration of the traced vibration; *A*_0_ and *A_t_* are the amplitudes of fundamental oscillation at the beginning and end of simulation, respectively. In terms of Equation (6), the Q factors of these four samples are then calculated to be 1463, 2520, 2321 and 4405, respectively. It is apparent from these four charts that the graphene sheet with smallest width and stress has much more energy loss in oscillation, while that with the largest width and stress has the least energy loss, and its Q factor is three times bigger than that of the former. The intermediate two also clearly show less amplitude diminishing, with Q factors of 2520 and 2321, respectively. Incidentally, the ripples on the margins of these amplitude curves occur due to the incoherent mixing of vibrations of two modes with different frequencies, which is called the beating phenomenon. Comparing the four results, it can be seen that enlargement of the width and stress could effectively improve the Q factor of graphene resonators.

For clarity, the impacts of the width and stress on the Q factor are therefore further studied individually. Figure 7e presents the relationship between the Q factor and the axial stress. As can be seen, the Q factor rises rapidly with increasing stress from 1463 at 10 GPa to 7989 at 50 GPa. Moreover, the Q factor is proportional to stress to the 2.5 power. In addition, widening the graphene sheets can also effectively improve the Q factor, as depicted in Figure 7f. For the 50 Å wide graphene sheet with a width–length ratio of 0.5, energy dissipations are quite violent, and the corresponding Q factor is calculated to be as low as 1463. When the width increases to 80 Å, the Q factor rises to 2321, but it does not continue to clearly rise with a larger width. The fitting of a power equation shows an exponent of about 0.12. As a result, increasing the tensile stress in graphene can greatly optimize the Q factor of graphene resonators, and it also enhances the sensitivity for mass measuring. Moreover, widening the graphene sheets with a width–length ratio near 0.8 considerably improves the Q factor; however, an excessive width seems to be useless for a further increase in Q value.

## 4. Conclusions

In conclusion, classical molecular dynamics simulations are adopted to investigate the impact of edge mode on mass sensing for a doubly clamped monolayer graphene resonator. The vibrations of edge mode were found to localize at the free edges and possess larger amplitude. Specifically, in the MD simulation for a 100 × 50 Å graphene sheet, the vibration amplitude of edge mode achieved 3.8 Å, nearly 2.5 times that of the fundamental mode. Moreover, the frequency gap of the two modes was calculated to be 32.6 GHz, which is irrelevant to the axial stress in graphene. Thus, considering that fundamental frequencies drop linearly with increasing centrally absorbed mass, while the frequencies of edge mode remain constant, a novel method of mass determination combining the two vibration modes was developed in order to diminish the interference of mutable stress in graphene sheets. More importantly, the MD results show that the vibration of edge mode broke the coherence of the mechanical fundamental oscillation, thus aggravating the energy dissipation and then resulting in a lower Q factor, which was as low as 1463 for the 100 Å × 50 Å graphene sheet with tensile stress of 10 GPa. However, increasing the stress in graphene can greatly increase the Q factor up to 7989 when an axial stress of 50 GPa is exerted. Moreover, appropriately widening the graphene sheets with a higher width–length ratio above 0.8 can double the Q factor, although excessive width does not contribute to an extremely high Q factor.
